# Benfotiamine, a Lipid-Soluble Derivative of Vitamin B_1_, Ameliorates the Carbohydrate Overload-Induced Mitochondrial Dysfunction in Fish *Megalobrama amblycephala* by Triggering the ULK1-Mediated Mitophagy

**DOI:** 10.1155/anu/7688386

**Published:** 2025-05-01

**Authors:** Ling Zhang, Chaofan He, Zishang Liu, Guangzhen Jiang, Wenbin Liu, Dingdong Zhang, Xiangfei Li

**Affiliations:** College of Animal Science and Technology, Nanjing Agricultural University, Nanjing, China

**Keywords:** carbohydrate metabolism disturbance, fish culture, mitochondrial dysfunction, mitochondrial nutrients, mitophagy

## Abstract

Compared with mammals, fish have a limited capability to utilize carbohydrates, thus generally suffering from metabolic disorders when offered carbohydrate-enriched diets. As a synthetic liposoluble derivative of vitamin B_1_, benfotiamine can alleviate the carbohydrate overload-induced mitochondrial dysfunction in fish, but the potential mechanisms have not been well explored. The present research was performed to unveil the molecular pathways through which benfotiamine benefits the mitochondrial function of a carp species *Megalobrama amblycephala*, which often exhibits metabolic disturbances. First, a control (C, 30% carbohydrate) group, a high-carbohydrate (HC, 43% carbohydrate) group, and a HC incorporating benfotiamine (1.425 mg/kg) group were conducted, respectively, in a 12-week feeding trial. Then, two in vitro studies were performed by using primary hepatocytes. In the first one, a media treatment, a high-glucose (HG) treatment, and a HG incorporating benfotiamine were designated, respectively. In the second one, a media group, a vehicle group, a HG group, and a HG + BL-918 (the agonist of UNC-51-like kinase 1 [ULK1]) group were adopted, respectively. The results indicated that HC/HG treatment resulted in mitophagy disorder by downregulating the phosphorylation of AMPK and ULK1 and the contents of proteins involved in the PTEN-induced putative kinase protein 1 (PINK1)-Parkin pathway. Mitochondrial dysfunction was also observed, as was indicative of the reduced activities of mitochondrial complex I, III, and SDH. However, benfotiamine treatment increased the contents of P-AMPK, P-ULK1, and the PINK1-Parkin pathway-related proteins as well as mitochondrial complex activities. In conclusion, benfotiamine could trigger the ULK1-mediated mitophagy to ameliorate the carbohydrate overload-induced mitochondrial dysfunction in fish.

## 1. Introduction

The abundant supply and low cost of carbohydrates contribute to making them the most economical source of energy for aquatic animals [1]. However, an overabundant consumption of this nutrient generally triggers metabolic disorders in aquatic species, thus causing great health concerns [2, 3]. Given this, the carbohydrate utilization capacity of fish has received extensive attention in the aquaculture industry. Mitochondria are critical for carbohydrate metabolism, and mitochondrial dysfunction has been demonstrated to be a key initiator of metabolic disorders like obesity, insulin resistance, and diabetes [4]. Previous studies indicate that mitochondrial function is closely linked to mitophagy, which determines mitochondrial homeostasis [5, 6]. Mitophagy, a process for clearing the damaged mitochondria, is triggered by the mitochondrial membrane potential (MMP), thus maintaining mitochondrial homeostasis and health [7]. Mitophagy starts with autophagosome formation, which is mediated by the core autophagic protein: UNC-51-like kinase 1 (ULK1) [8]. Upon mitophagy, ULK1 is upregulated and translocated to fragmented mitochondria [9], where it interacts with the FUN14 domain-containing protein 1 (FUNDC1) and enhances the binding of FUNDC1 to light chain 3 (LC3), thereby targeting mitochondria to autophagosomes [10]. Then mitophagy is mediated by several pathways with the PTEN-induced putative kinase protein 1 (PINK1)-E3 ubiquitin ligase (Parkin) pathway being the most mature and critical one studied in eukaryotic cells [11]. Recently, several studies suggest that mitophagy disorder is the mainspring of mitochondrial dysfunction in fish. For example, Dai et al. [12] have proven that the cadmium exposure-induced mitochondrial dysfunction in zebrafish (*Danio rerio*) is associated with oxidative stress and mitophagy. Séité et al. has also found that feeding the methionine-deficient diets triggered the PINK1/Parkin pathway-mediated mitophagy in rainbow trout (*Oncorhynchus mykiss*), thereby resulting in mitochondrial defects [13]. In addition, carbonylcyanide-3-chlorophenylhydrazone caused dysregulated mitochondrial homeostasis in blunt snout bream (*Megalobrama amblycephala*), as was manifested by an increase in mitochondrial oxidative stress and mitophagy along with a disruption of mitochondrial function [5]. All these literature suggest that mitophagy and mitochondrial function are closely linked in fish. They also highlight the importance to develop nutritional interventions that could promote mitophagy in the treatment of mitochondrial dysfunction in aquatic species.

To date, several research have established that dietary supplementation of mitochondrial nutrients is highly beneficial in the treatment of type 2 diabetes. Mitochondrial nutrients are a group of micronutrients that either are mitochondrial components or can influence both the structure and function of mitochondria, thereby protecting them from oxidative damage and dysfunction [14]. They can be classified into three main categories, including antioxidants (like vitamin E, lipoic acid, coenzyme Q, and glutathione), cofactors and their precursors (like the B-complex vitamins, lipoic acid, and coenzyme Q), and energy enhancers and others (like choline, carnitine, and creatine) [15]. As a lipid-soluble analog of thiamine, benfotiamine is highly bioavailable and has shown significant efficacy in improving the pathology caused by diabetic symptoms [16, 17]. For instance, it could alleviate mitochondrial dysfunction and reduce the oxidative damage and inflammation in mouse [18]. Furthermore, it can inhibit the biochemical pathways implicated in the pathogenesis of hyperglycemia in the retinas of diabetic animals [19], thus attenuating diabetes and its related complications [20]. However, the existing research has mainly concentrated on mammals. Recently, it has been demonstrated that benfotiamine facilitates mitochondrial biogenesis, antioxidant capacity, and mitochondrial function in fish fed high-carbohydrate (HC) diets [21, 22]. However, the underlying molecular mechanisms for achieving these benefits remain poorly elucidated.

This study intended to unveil the molecular mechanisms through which benfotiamine benefits the mitochondrial function of fish both in vivo and in vitro by using an herbivorous carp species *M. amblycephala*, which struggles considerably from metabolic disorders in practical aquaculture as a result of using high nonprotein energy diets [23, 24]. Previously, this species has been considered as a suitable model for investigating glucose metabolism disorders in fish [25, 26]. The present work mainly focuses on mitophagy given its close relationship with mitochondrial function. Moreover, the probable functions of ULK1 in the aforementioned processes were also identified. The results presented here could partly reveal the molecular targets of benfotiamine, as might advance the development of precise nutritional interventions to promote the carbohydrate utilization by aquatic animals.

## 2. Materials and Methods

### 2.1. Ethics Statement

Fish were treated following the protocols of the Care and Use of Nanjing Agricultural University (SYXK [Su] 2011-0036).

### 2.2. The In Vivo Study

A basal mixture was prepared, including 8% fish meal, 26% soybean meal, 17% rapeseed meal, 17% cottonseed meal, 2% fish oil, 2% soybean oil, 1.8% calcium biphosphate, and 1.2% mineral and vitamin premix. Then, a total of three experimental diets were manufactured, including a control (C, 30% nitrogen-free extract) one, a HC (43% nitrogen-free extract) one, and a HC incorporating benfotiamine (RongZhen, China) (1.425 mg/kg, HCB) one. Dietary carbohydrate levels were achieved by adding different contents of corn starch (12% for the C diet and 25% for the HC diet) and were compensated by microcrystalline cellulose. The premix was detailed by Zhang et al. [22], and the benfotiamine does and dietary carbohydrate levels were referred to Xu et al. [21, 22]. Feed formulation and nutrient contents were shown in Table 1. A total of 240 fish *M. amblycephala* (average around 19.7 g) were randomly distributed into 12 tanks (volume: 200 L) with a density of 20 fish/tank. Following a 2-week domestication period, fish were provided with the three experimental diets (4 cages/diet) and were fed three times daily for 12 weeks with visual satiety as a feeding guideline. The feed amount was monitored during the feeding trial to calculate the feed utilization parameters.

Prior to sampling, a 24-h fasting was adopted to empty the intestinal contents. Then, fish were rapidly anesthetized with 3-aminobenzoic acid ethyl ester methanesulfonate (MS-222, 100 mg/mL, E10521, Sigma–Aldrich, China). Subsequently, four fish were selected from each cage and were weighed at random. Then, blood was withdrawn from caudal peduncle and was centrifuged to collect the supernatant. The liver was rapidly separated at a low temperature, and the tissue samples were separated into centrifuge tubes for further analysis. Next, the livers of four fish within each treatment were prepared into cell suspension for the determination of MMP. Additionally, the liver of one fish from each test group was rapidly removed and preserved in 2.5% glutaraldehyde (111-30-8, Merck, China) for the transmission electron microscopy (TEM) analysis.

### 2.3. The In Vitro Study

The primary hepatocytes were isolated from the livers of *M. amblycephala* following the protocols described previously [22]. Briefly, blood was withdrawn from the healthy and untreated fish. Then the abdomen was dissected, and the liver tissue was collected with the fish cleansed with an alcohol spray during this procedure. Then, the minced liver was then digested in sterile trypsin (25200056, Gibco, China). Hepatocyte precipitates were obtained by filtering the above cell suspension and then mixing it with PBS followed by a centrifugation. After treatment with the DMEM/F12 medium (21041025, Gibco, China), a cell suspension with a density of 1 × 10^5^ was obtained. After seeding into a 6-well plate, hepatocytes were incubated in a full-growth medium for 48 h and were serum starved for 24 h before being cultured in the treatment group for another 24 h.

A total of two in vitro studies were performed. In the first one, cells were treated with media, high-glucose (HG) (80 mM glucose), and HG incorporating different doses of benfotiamine, respectively [22]. The last three groups were labeled as HGB1 (1.459 μg/mL), HGB2 (2.918 μg/mL), and HGB3 (5.836 μg/mL). In the second one, cells were treated with media, vehicle, HG, and HG incorporating BL-918 (the agonist of ULK1, CAS: 2101517-69-3, MedChemExpress, China). Dimethyl sulfoxide (DMSO) was used as the solvent in this experiment to achieve the final concentration of BL-918 at 5 μM/mL [27].

### 2.4. Measurement Procedures

#### 2.4.1. Analysis of Feed Components

According to the method of AOAC [28], the proximate composition of experimental feeds was analyzed. Briefly, the samples were ground into powder and dried to a constant weight in an oven at 105°C for the determination of moisture content. Subsequently, the crude protein content was determined using a fully automatic Kjeldahl nitrogen analyzer (K1100, Hanon, China). The crude lipid content was calculated after extracting the lipid from the samples by Soxhlet extraction method using a fully automatic fat analyzer (XT10i, ANKOM, China). The crude ash content was determined using a muffle furnace (XL-2A, Zhuochi Instrument Co., Ltd., China) with the samples calcined at 550°C for 6 h. The crude fiber content was analyzed using an automated fiber analyzer (ANKOM A2000i, ANKOM Technology, USA) after an acid and alkali treatment. The total energy was determined by an oxygen bomb calorimeter (IKAC6000, Shenhua Biotechnology Co., Ltd, China).

#### 2.4.2. Plasma Metabolites and Hepatic Glycogen and Lipid Contents

Triglyceride (TG) and cholesterol (CHO) contents in plasma were quantified following the detailed scheme of Asset et al. [29]. Both hepatic glycogen and crude lipid contents were quantified following the methods documented by Seifter et al. [30] and Folch et al. [31], respectively.

#### 2.4.3. The TEM Analysis

The livers fixed in the 2.5% glutaraldehyde phosphate buffer were removed and immersed in 1% osmium tetroxide for 65 min. Next, the samples were dehydrated through ethanol and were embedded in epoxy resin. Then, the embedded tissue blocks were ultrathin-sectioned and double-stained with 3% uranyl acetate-lead citrate. Finally, the tissues were photographed with a TEM (JEOL, JEM-1230) at 80 KV.

#### 2.4.4. MMP and Mitochondrial Function-Related Enzyme Activities

Hepatocytes were stained by tetrechloro-tetraethylbenzimidazol carbocyanine iodide (JC-1) [32]. Firstly, the liver tissues were removed and chopped with a scalpel. Then both collagenase (9001-12-1, Sigma–Aldrich, China) and trypsin (25200-056, Gibco, China) were used to digest the liver. And the cell suspension was then filtered using cell sieves (100 μm) to remove larger cell clusters. Subsequently, the cell filtrate was centrifuged (500 g, 10 min) to obtain the cell precipitate. Then, the above cell precipitate/primary hepatocytes were resuspended in the formulated fluorescent probe JC-1 solution (3520-43-2, Yeasen, China). After incubation for 20 min, the flow cytometry was utilized to detect the MMP. Furthermore, mitochondria from liver/hepatocytes were isolated using a mitochondrial extraction kit (HY-K1061, MedChemExpress, China). The activities of NADH–ubiquinone oxidoreductase (Complex I), succinate–ubiquinone oxidoreductase (Complex II), ubiquinone–ferricytochrome–c oxidoreductase (Complex III), cytochrome c oxidase (complex IV), F1F0–ATP synthase (Complex V), succinate dehydrogenase (SDH), and citrate synthase (CS) were all determined by ELISA assay according to the instructions of assay kits (Jiangsu Meimian, China). Finally, the activities of above enzymes were calculated from the standard curve for each sample, respectively.

#### 2.4.5. Western Blotting

A 0.1 g liver sample was ground with liquid nitrogen and was added with 500 μL of total protein extraction buffer (radioimmunoprecipitation assay lysis buffer:phenylmethylsulfonyl fluoride = 1000:1). After a full vortex centrifugation, the supernatant was retained. This process was repeated twice. The protein concentration was uniformed by dilution, and then 5× protein denaturation buffer (SDS 5×) was added and denaturated at 95°C for 10 min. An electrophoresis was adopted to isolate proteins from the samples using the 4%–20% SDS-polyacrylamide gel (36256, Yeasen, China). In addition, polyvinylidene fluoride (PVDF) membranes were used for the protein-transfer step, which were then immersed in the primary antibodies followed by a three-time membrane wash with tris-buffered saline with Tween-20 (TBST). Then, the membranes were immersed in the secondary antibody (BA1054, Boster, China) for 2 h and sealed in a fast blocking solution (36122ES76, Yeasen, China) for 20 min. After the antigen–antibody immunoreaction, the membranes were immersed in enhanced chemiluminescent substrate (ECL, 36208, Yessen, China) and photographed with ChemDoc Chemiluminescent Imager (Bio-Rad).

The primary antibodies are as follows: anti-tubulin (#AF7010, Affinity, China), anti-total-AMPK α (#2532, CST, USA), anti-phospho-AMPK α (#2535, CST, USA), anti-total-ULK1 (20986-1-AP, Proteintech, China), anti-phosphor-ULK1 (AP0760, Abclonal, China), anti-PINK1 (#DF7742, Affinity, China), anti-Parkin (#A11172, Abclonal, China), anti-P62 (sequestosome 1, #ab91526, Abcam, UK), anti-LC3B (#2775s, CST, USA), and anti-TIM23 (#11123-1-AP, Proteintech, China).

### 2.5. Data Analysis

The data were analyzed using SPSS statistics 22.0. In the feeding experiment and the second in vitro study, the significant differences between groups were analyzed by Student's *t*-test. In the first in vitro study, Student's *t*-test was adopted to analyze the data between the media and the HG group, while one-way ANOVA was adopted to assess the results among different doses of benfotiamine in the HG treatments by using the Tukey's multiple range test. Meanwhile, the type of significance (linear, quadratic, or cubic) was further tested by the orthogonal polynomial contrasts. All results were presented as mean ± SEM of four biological replicates. Bars assigned with different superscripts are significantly different (*⁣*^*∗*^*p* < 0.05; *⁣*^*∗∗*^*p* < 0.01; *⁣*^*∗∗∗*^*p* < 0.001).

## 3. Results

### 3.1. The In Vivo Study: Investigating the Effects of Benfotiamine in a Feeding Trial

#### 3.1.1. Growth Performance, Plasma Metabolites, and Hepatic Glycogen and Lipid Contents

**Figure 1:**
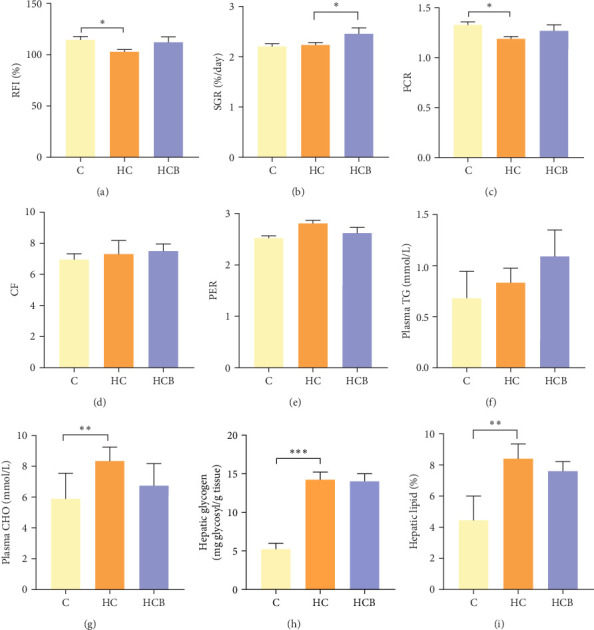
Growth performance, plasma metabolic parameters, and hepatic glycogen and lipid contents of *M. amblycephala* in the feeding trial. (A–E) Growth performance-related parameters, (F) plasma TG, and (G) CHO as well as (H, I) hepatic glycogen and lipid contents. Relative feed intake (RFI, %) = feed consumption (g)/*W*_t_ (g) × 100. Specific growth rate (SGR, %/day) = (Ln*W*_t_ – Ln*W*_0_) × 100/*T*, where *W*_0_ and *W*_t_ are the initial and final body weights and *T* is the culture period in days. Feed conversion ratio (FCR) = feed consumption (g)/fish weight gain (g). CF = (*W*/*L*^3^) × 100, where *W* is body weight (g) and *L* is body length (cm). Protein efficiency ratio (PER) = fish weight gain/total protein fed. *⁣*^*∗*^*p* < 0.05; *⁣*^*∗∗*^*p* < 0.01; *⁣*^*∗∗∗*^*p* < 0.001.

Condition factor (CF), protein efficiency ratio (PER), and plasma TG levels all exerted little difference (*p* > 0.05) among all the groups (Figure 1). However, hepatic glycogen and lipid contents and plasma CHO concentrations were found significantly (*p* < 0.05) higher in the HC group in comparison to the C group, while an opposite result was noted in relative feed intake (RFI) and feed conversion ratio (FCR). Furthermore, the HCB group considerably enhanced the specific growth rate (SGR) compared to the C group (*p* < 0.05).

#### 3.1.2. TEM Analysis

**Figure 2:**
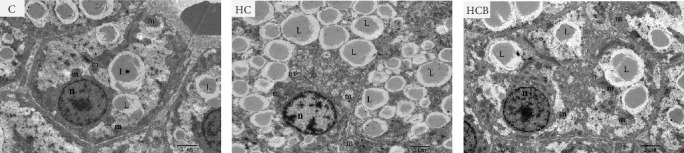
The morphological structure of the liver of *M. amblycephala* in the feeding trial. L, lipid droplets; m, mitochondria; n, nucleus.

The C group showed a normal nuclear structure and mitochondrial size, whereas the nuclear components of cells were degraded with increased lipid droplets and swollen and whitish mitochondria in the HC group. Compared with the HC group, the supplementation of benfotiamine almost restored the normal hepatic structure with a normal mitochondrial morphology and size noticed (Figure 2).

#### 3.1.3. Mitophagy

**Figure 3:**
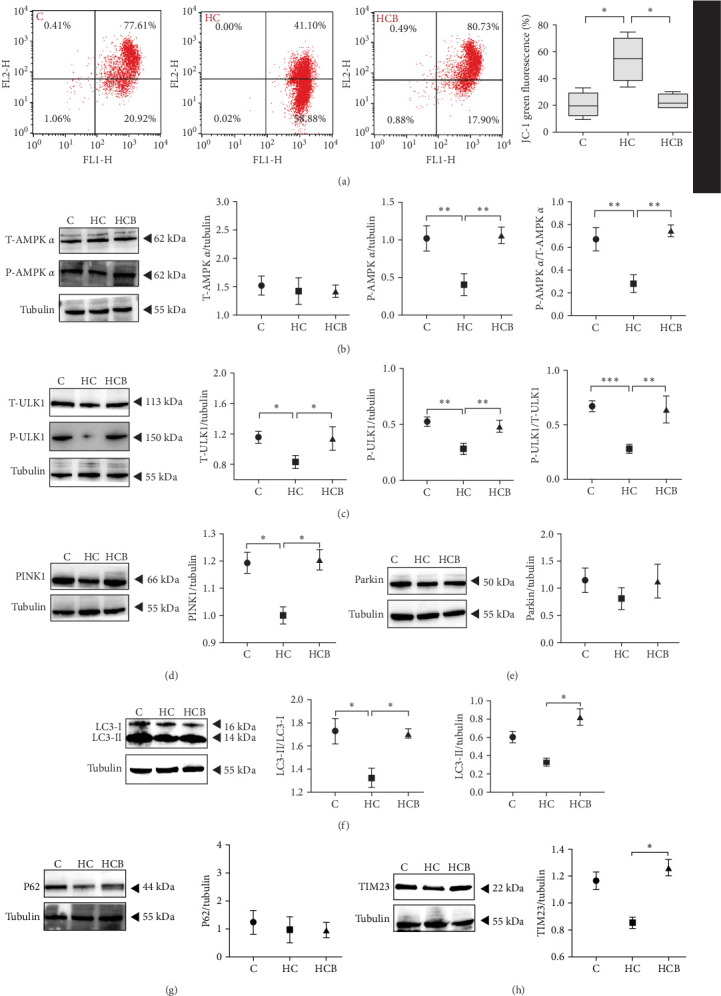
Benfotiamine alleviates mitophagy disorder in the liver of *M. amblycephala* in the feeding trial. (A) JC-1 and (B–H) mitophagy-related protein expressions. *⁣*^*∗*^*p* < 0.05; *⁣*^*∗∗*^*p* < 0.01; *⁣*^*∗∗∗*^*p* < 0.001.

The HC group showed a considerably higher monomer fluorescence with a low MMP than the C group, while benfotiamine supplementation reversed this (*p* < 0.05) (Figure 3A). However, the protein expressions of T-AMPK α, Parkin, and P62 all exerted little difference (*p* > 0.05) among all the groups (Figure 3B,E,G). And feeding the HC diet significantly (*p* < 0.05) decreased the protein contents of P-AMPK α, T-ULK1, P-ULK1, and PINK1 and the ratios of P-AMPK α/T-AMPK α, P-ULK1/T-ULK1, and LC3-Ⅱ/LC3-I in comparison to the C group. However, the HCB group significantly (*p* < 0.05) reversed these changes compared with the HC group (Figure 3B–D,F,H).

#### 3.1.4. Mitochondrial Function

**Figure 4:**
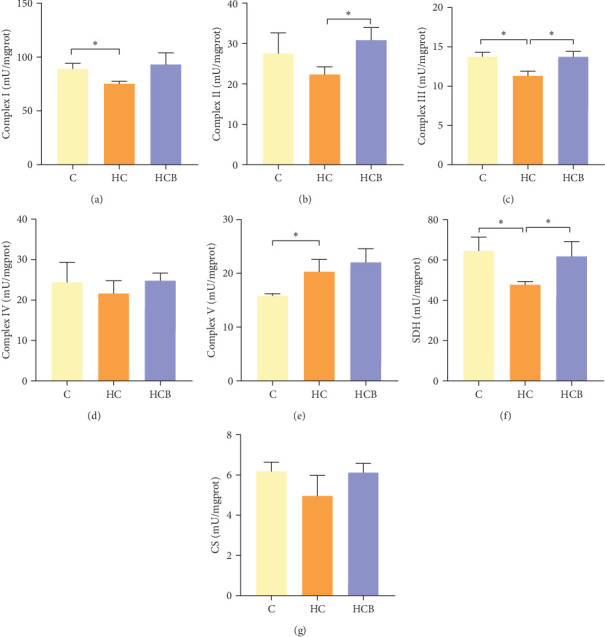
Benfotiamine alleviates mitochondrial dysfunction in the liver of *M. amblycephala* in the feeding trial. (A–E) The activities of mitochondrial complex IV, CS (F), and SDH (G). *⁣*^*∗*^*p* < 0.05.

The HC group obtained considerably (*p* < 0.05) lower activities of complex Ⅰ and Ⅲ and SDH compared with the C group (Figure 4A,C,F). However, benfotiamine supplementation significantly (*p* < 0.05) elevated the activities of complex Ⅱ and Ⅲ and SDH than the HC group (Figure 4B,C,F). Additionally, the activities of complex Ⅳ and CS both exerted little difference (*p* > 0.05) among all the groups (Figure 4D,G).

### 3.2. The In Vitro Study (Exp. 1): Validating the Effects of Benfotiamine

#### 3.2.1. Mitophagy

**Figure 5:**
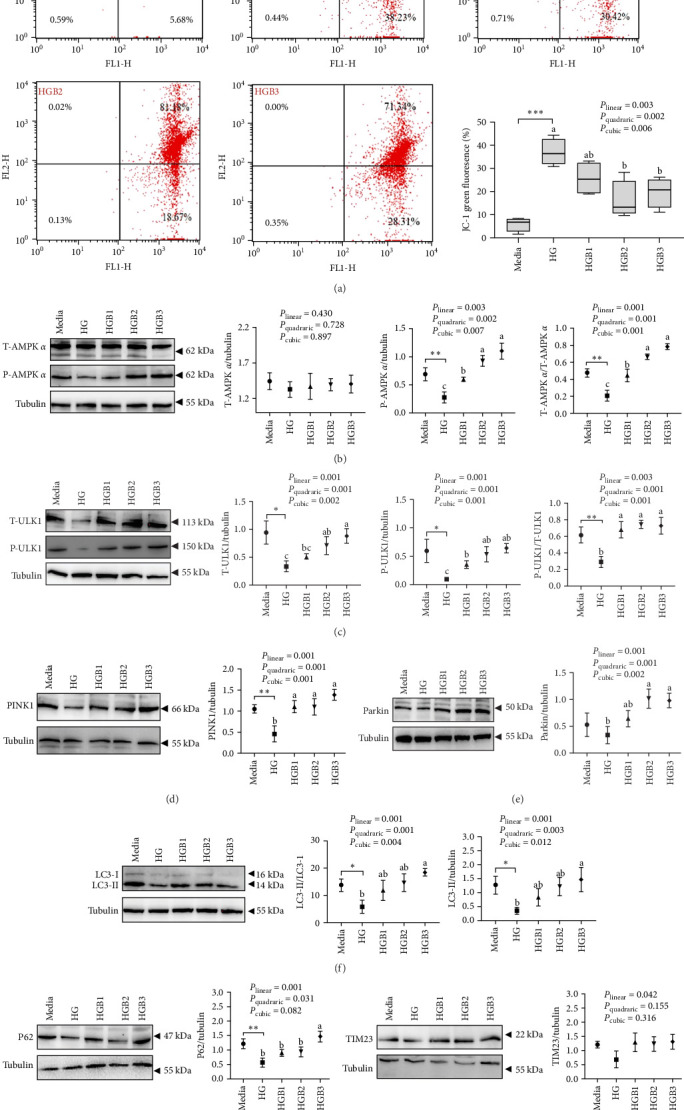
Benfotiamine reverses mitophagy disorder in the primary hepatocytes of *M. amblycephala* cultured with high glucose. (A) JC-1 in primary hepatocytes and (B–H) mitophagy-related protein expressions. *⁣*^*∗*^*p* < 0.05, *⁣*^*∗∗*^*p* < 0.01, *⁣*^*∗∗∗*^*p* < 0.001, compared between the media and the HG group. abc, compared among different benfotiamine levels in the HG groups. Bars assigned with different letters are significantly different (*p* < 0.05).

The HG group obtained a significantly higher monomer fluorescence with a low MMP (Figure 5A), and significantly lower protein contents of P-AMPK α, T-ULK1, P-ULK1, PINK1, LC3-II, and P62 and the ratios of P-AMPK α/T-AMPK α, P-ULK1/T-ULK1, and LC3-II/LC3-I (Figure 5B–D,F,G) than those of the C group (*p* < 0.05). However, benfotiamine supplementation significantly (*p* < 0.05) elevated the protein contents of P-AMPK α, T-ULK1, P-ULK1, PINK1, Parkin, LC3-II, and P62 and the ratios of P-AMPK α/T-AMPK α, P-ULK1/T-ULK1, and LC3-II/LC3-I (Figure 5B–G) with a linear, quadratic, and cubic significance observed.

#### 3.2.2. Mitochondrial Function

**Figure 6:**
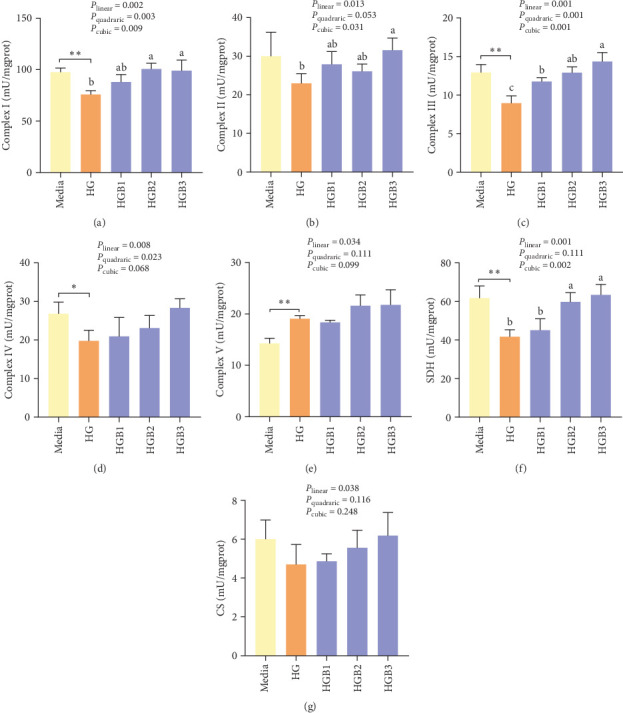
Benfotiamine reverses mitochondrial dysfunction in the primary hepatocytes of *M. amblycephala* cultured with high glucose. (A–E) The activities of mitochondrial complex IV, (F, G) CS, and SDH. *⁣*^*∗*^*p* < 0.05, *⁣*^*∗∗*^*p* < 0.01, compared between the media and the HG group. abc, compared among different benfotiamine levels in the HG groups. Bars assigned with different letters are significantly different (*p* < 0.05).

The HG group obtained significantly (*p* < 0.01) lower activities of complex Ⅰ, Ⅲ, and Ⅳ and SDH compared to the C group (Figure 6A,C,D,F). The HGB groups significantly (*p* < 0.05) elevated the activities of complex Ⅰ, Ⅱ, and Ⅲ and SDH compared with the HG group with a linear, quadratic, and cubic significance observed in the activities of complex Ⅰ and Ⅲ (Figure 6A–C,F). However, only a linear significance was observed in the activities of complex Ⅱ, Ⅳ, and Ⅴ as well as SDH and CS (*p* < 0.05).

### 3.3. The In Vitro Study (Exp. 2): Validating the Molecular Targets of Benfotiamine

#### 3.3.1. Mitophagy

**Figure 7:**
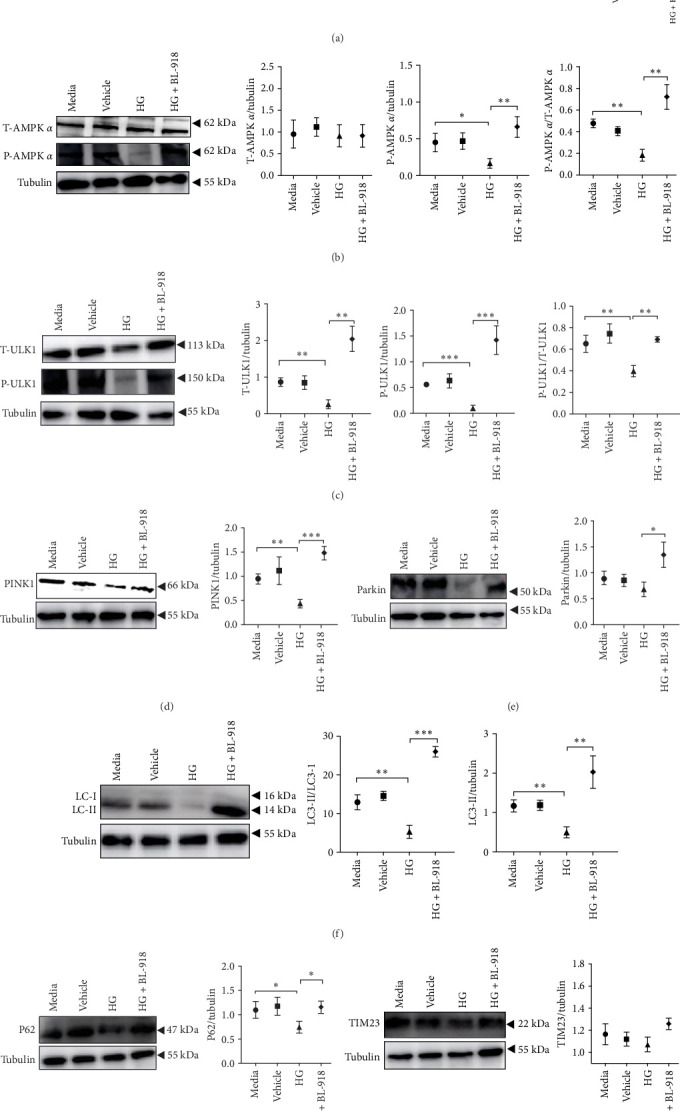
The effects of ULK1 agonist treatment on mitophagy in the primary hepatocytes of *M. amblycephala* cultured with high glucose. (A) JC-1 and (B–H) mitophagy-related protein expressions. *⁣*^*∗*^*p* < 0.05; *⁣*^*∗∗*^*p* < 0.01; *⁣*^*∗∗∗*^*p* < 0.001.

The HG group presented significant higher monomer fluorescence with lower MMP than that of the C group, while the HG + BL-918 group reversed this (Figure 7A) (*p* < 0.01). Additionally, the HG group obtained significant (*p* < 0.05) lower protein expressions of P-AMPK α, T-ULK1, P-ULK1, PINK1, and LC3-II and the ratios of P-AMPK α/T-AMPK α, P-ULK1/T-ULK1, and LC3-II/LC3-I compared with the C group (Figure 7B–D,F). However, the HG + BL-918 group significantly (*p* < 0.05) elevated the protein expressions of P-AMPK α, T-ULK1, P-ULK1, PINK1, Parkin, LC3-II, and P62 and the ratios of P-AMPK α/T-AMPK α, P-ULK1/T-ULK1, and LC3-II/LC3-I, compared with the HG group (Figure 7B–F).

#### 3.3.2. Mitochondrial Function

**Figure 8:**
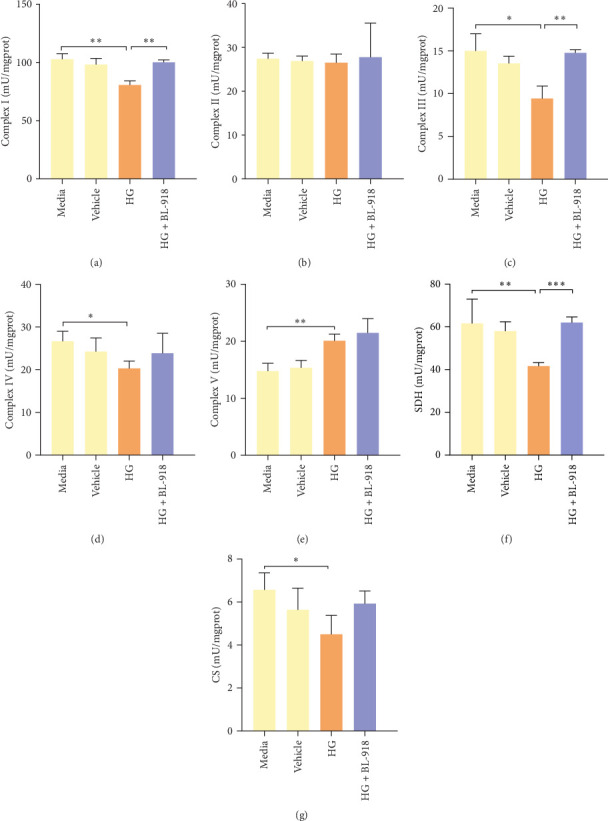
The effects of ULK1 agonist treatment on mitochondrial function in the primary hepatocytes of *M. amblycephala* cultured with high glucose. (A–E) The activities of mitochondrial complex IV, (F, G) CS, and SDH. *⁣*^*∗*^*p* < 0.05; *⁣*^*∗∗*^*p* < 0.01; *⁣*^*∗∗∗*^*p* < 0.001.

The activities of complex I, III, and Ⅳ, SDH, and CS of the HC group were significantly higher than those of the C group (Figure 8A,C,D,F,G). Compared with the C group, the HG + BL-918 group significantly (*p* < 0.05) upregulated the activities of complex Ⅰ and Ⅲ and SDH (Figure 8A,C,F).

## 4. Discussion

The HC feeding resulted in a decreased RFI and FCR but increased plasma CHO concentration and hepatic glycogen and lipid contents in *M. amblycephala* in this study. HC diet generally leads to a reduced feed palatability, as could accelerate the satiety of aquatic animals, thus leading to a low feed consumption and high feed efficiency [33]. In addition, benfotiamine supplementation may have increased the activities of intestinal digestive enzymes and facilitated nutrient absorption [34], thereby resulting in a significant increase in SGR. Additionally, a marked increase in total plasma CHO levels was also observed by HC feeding compared to the C group. Typically, HC feeding could stimulate the synthesis of fatty acids, which in turn increased plasma TG and CHO levels [35].

In this study, swollen mitochondria were found in the HC group. Meanwhile, the MMP was also found to be lower in the HC/HG group. This could result from the overproduction of reactive oxygen species (ROS) [36], since one of the key events coupled with ROS generation is mitochondria swelling [36, 37]. In comparison, benfotiamine supplementation considerably restored the MMP, as may be explained by its efficacy in reducing mitochondrial oxidative stress, thus protecting against MMP collapse [22]. Furthermore, MMP may also trigger the PINK1-Parkin pathway-mediated mitophagy [38]. To further determine whether mitochondrial oxidative stress induced by high carbohydrates/glucose could affect mitophagy process in fish, the expressions of mitophagy-related proteins were measured in the present study both in liver and primary hepatocytes. The HC treatment significantly downregulated the protein expression of P-AMPK, T-ULK1, and P-ULK1 and the ratios of P-AMPK α/T-AMPK α and P-ULK1/T-ULK1. The same results were also observed in the HG group, suggesting that HC/HG treatment may inhibit the mitophagy pathway in fish. It has been reported that AMPK is a cellular stress-sensing protein that regulates a variety of metabolic processes including mitophagy [39] through a direct activation on metabolism-related proteins or an indirect influence on gene expression [40]. The decreased P-AMPK expression could be attributed to the HG content, which usually results in an increased oxidative phosphorylation rate followed by an enhanced ATP production [41]. This could consequently increase the intracellular ATP content, which generally inhibits the AMPK activity in fish [42]. This might in turn inhibit the AMPK-mediated mitophagy signaling. Generally, the inhibited AMPK activity may result in the inactivation of ULK1, which is a downstream target of AMPK. In addition, the HC/HG treatment remarkably decreased the protein levels of PINK1, LC3-Ⅱ, P62, and TIM23 and the LC3-Ⅱ/LC3-Ⅰ ratio, as also indicated a reduced mitophagy level. Generally, upon the disintegration of the MMP, PINK1 aggregates stably on the mitochondrial membrane and activates the translocation of Parkin from the cytoplasm into the mitochondria. Then, LC3-I generates LC3-II via ubiquitin binding to phosphatidylethanolamine, which is then mobilized to autophagosomes [43]. Meanwhile, the mitophagy adaptor P62 identifies LC3-II to foster autophagosome generation [44]. Additionally, the expression of TIM23 is also a reliable indicator of the mitophagy activity, since the protein level of TIM23 is generally reduced with the removal of damaged mitochondria through mitophagy [45]. The depletion of ULK1 in the HC/HG group may also lead to the inactivation of Parkin and the inhibited downstream mitophagy events [39]. In addition, the increased P62 expression generally inhibits mitochondrial flux and degradation in lysosomes [46], as is obviously contrary to the results obtained in this study. This inconsistence is justifiable, since the nuclear factor erythroid 2-related factor 2/Kelch-like ECH-associating protein 1 (Nrf2/Keap1) pathway functions in the cellular defense system to endogenous or exogenous oxidative stress [47]. In fact, P62 could activate Nrf2 through Keap1 degradation, and Nrf2 itself could trigger P62 expression [48]. Therefore, the increased P62 expression might indicate an oxidative stress resistance to combat stress rather than an indication of the inhibited mitophagy. Additionally, MMP collapse is a prerequisite for the PINK1-Parkin pathway activation and mitophagy. However, surprisingly, the MMP loss did not induce hepatic mitophagy in this study but instead inhibited it. Two possibilities might be advanced to explain this result: (1) the HC/HG treatment inhibited the mitophagy level [5], as in turn prevents the removal of fragmented mitochondria and also causes the inability to restore the MMP, and (2) the loss of MMP is also a landmark event in the early stages of apoptosis [45]. Therefore, the reduction of MMP in the HC/HG group may also reflect an onset of apoptosis by HC/HG treatment. Furthermore, in this study, benfotiamine supplementation attenuated the adverse effects of high carbohydrate/glucose on the MMP and mitophagy in fish, as was evidenced by the increased protein expressions of P-AMPK α, ULK1, PINK1, LC3-II, and TIM23 and the increased ratios of P-AMPK α/T-AMPK α and LC3-II/LC3-I ratios. This may result from the alleviation of mitochondrial oxidative stress by benfotiamine, as in turn restores the level of mitophagy [5].

To determine whether the alterations in the degree of mitophagy would affect mitochondrial function, the activities of mitochondrial complexes, SDH, and CS were further determined. The results indicated that the HC/HG treatment reduced the activities of complex Ⅰ and Ⅲ and SDH but decreased that of complex V, suggesting a mitochondrial dysfunction. This may result from the inhibition of mitophagy caused by excessive carbohydrate/glucose load, since mitophagy is the main pathway to clear dysfunctional mitochondria, thereby sustaining mitochondrial function fitness [49]. Consistent with this, it has been proved that an inhibition of the PINK1/Parkin-mediated mitophagy exacerbates the mitochondria dysfunction in diabetic mice, thereby resulting in decreased mitochondrial function [49]. The elevated complex V activity is probably resulted from the enhanced energy uptake in the HC/HG groups. In fact, complex V can drive the generation of ATP [50]. High-energy expenditure generally results in an increased oxidative phosphorylation rate in mitochondria [41], which in turn augments the activity of complex V [51]. However, benfotiamine increased the activities of complex Ⅰ, Ⅱ, and Ⅲ and SDH, reflecting an enhanced mitochondrial function by benfotiamine. According to a previous study, benfotiamine can impede the oxidative stress in mitochondria by scavenging free radicals and suppressing the generation of oxidants, thereby increasing the activities of mitochondrial respiratory enzymes [52]. The enhancement of mitophagy by benfotiamine could also be advanced to interpret this result. Overall, all the above results verified that benfotiamine could ameliorates the HC/HG treatment-induced mitophagy disorder and mitochondrial dysfunction in fish.

Previous findings from this work revealed that benfotiamine could alleviate the carbohydrate overload-induced mitophagy disorder presumably via stimulating the ULK1 protein expression. In order to further confirm this, the ULK1 agonist was further used in vitro. BL-918 is considered a powerful activator of ULK1, which could activate the ULK complex, and ultimately triggers cellular protective autophagy [53]. Consistent with the above results, the HG treatment caused mitophagy disorder and mitochondrial dysfunction, while the BL-918 treatment exerted similar positive benefits as benfotiamine. Furthermore, the BL-918 treatment remarkably enhanced mitochondrial function compared with the HG group. This further confirmed that the enhancement of mitophagy could improve mitochondrial function in fish. Overall, the results indicated that benfotiamine mitigated the HC-/HG-induced inhibition of mitophagy through the activation of ULK1, thus facilitating the elimination of damaged mitochondria, as might ultimately enhance mitochondrial function.

## 5. Conclusions

**Figure 9:**
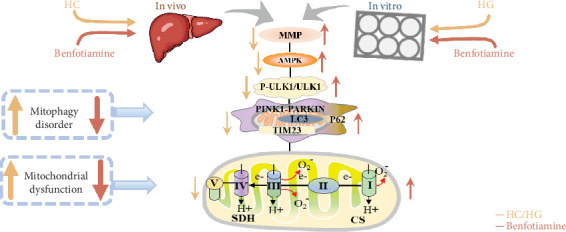
Benfotiamine ameliorates the carbohydrate overload-induced mitochondrial dysfunction in fish *M. amblycephala* by triggering the ULK1-mediated mitophagy.

In conclusion, a HC/HG treatment inhibited the protein level of ULK1 in fish *M*. *amblycephala*, as consequently led to mitophagy disorder and mitochondrial dysfunction. Benfotiamine supplementation promoted mitophagy through the activation of ULK1, as ultimately improved mitochondrial function (Figure 9).

## Figures and Tables

**Table 1:** Formulation and proximate composition of the diets.

Formulation (%)	C	HC	HCB
Basal mixture	75.00	75.00	75.00
Corn starch	12.00	25.00	25.00
Benfotiamine (mg/kg)	0.00	0.00	1.425
Microcrystalline cellulose	13.00	0.00	0.00

**Proximate composition (% air-dry basis)**			

Moisture	6.87	6.76	6.89
Crude lipid	5.66	5.55	5.77
Ash	8.31	8.23	8.19
Crude protein	29.79	29.91	30.02
Crude fiber	17.01	6.29	6.21
Nitrogen-free extract	32.36	43.26	42.92
Gross energy (MJ/kg)	19.12	19.31	19.27

## Data Availability

The data that support the findings of this study are available on request from the corresponding author. The data are not publicly available due to privacy or ethical restrictions.
